# Cardiac Catheterization in Assessment and Treatment of Kawasaki Disease in Children and Adolescents

**DOI:** 10.3390/children6020032

**Published:** 2019-02-21

**Authors:** Hitesh Agrawal, Athar M. Qureshi

**Affiliations:** 1 Invasive Cardiac Imaging and Interventional Catheterization Laboratory, Le Bonheur Children’s Hospital, The University of Tennessee Health Sciences Center, Memphis, TN 38103, USA; 2 C.E. Mullins Cardiac Catheterization Laboratories, The Lillie Frank Abercrombie Section of Cardiology, Texas Children’s Hospital, Baylor College of Medicine, Houston, TX 77030, USA; axquresh@texaschildrens.org

**Keywords:** Kawasaki disease, fractional flow reserve (FFR), intravascular ultrasound (IVUS), angioplasty, stenting

## Abstract

Cardiac catheterization has become a promising tool to assess and treat coronary artery lesions in patients with Kawasaki disease. Significant coronary artery lesions can now be treated via transcatheter route even in small children. Further development and miniaturization of this technology will help to promote widespread use to the benefit of small children suffering from coronary artery disease. The role of diagnostic and interventional coronary artery procedures in children and adolescents are discussed in this article.

## 1. Introduction

Kawasaki disease is an acute vasculitis affecting small to midsize arteries and is one of the leading cause of acquired cardiovascular disease in children. Non-invasive imaging with electrocardiograms, echocardiograms, stress tests, computed tomography (CT) angiograms, and cardiac magnetic resonance imaging (MRI) are primarily used to diagnose coronary abnormalities and assess their physiologic significance. However, in some cases, cardiac catheterization with invasive anatomic and functional testing with intravascular ultrasound (IVUS), fractional flow reserve (FFR) measurement, and optical coherence tomography (OCT) are promising tools which can guide clinical decision making [1]. In select cases, various treatments, including angioplasty, stenting, and rotational ablation can be performed in the same setting if indicated. Such interventions are becoming increasingly common in children and pediatric interventionalists need to be familiar with these techniques. We discuss the role of coronary artery angiography, FFR, IVUS, OCT, angioplasty, stenting, and rotational ablation in children and adolescents with Kawasaki disease in this review. The reader is also encouraged to refer to the recent comprehensive American Heart Association Scientific Statement regarding recommendations and evidence for performing these procedures [2].

## 2. Coronary Angiography

Coronary angiography is generally not indicated in the acute phase of illness. For longer term follow up, however, coronary angiography can be extremely beneficial for periodic surveillance of significant coronary artery aneurysms and the coronary arteries. Coronary angiography is also indicated for any patient with inducible defects, depressed ventricular function, or other signs of coronary artery compromise. Despite its invasiveness, coronary angiography is superior to other imaging modalities (i.e., CT scan), particularly in young infants with fast heart rates, which limits the utility of CT scan imaging. Coronary angiography delineates the luminal diameter of the affected coronary artery segment in relation to the normal vessel, degree of stenosis, calcified areas, and aneurysm size. In addition, the distal coronary vasculature can be evaluated, which can be difficult to do with other imaging modalities. Anatomic mapping with coronary angiography is an important step prior to embarking on provocative functional testing or transcatheter interventions, or surgical bypass grafts. For pre-operative planning, angiograms of the subclavian arteries or internal mammary arteries selectively are important for surgical planning, should internal mammary arteries need to be used for coronary artery bypass in the future. Angiography is again helpful to assess the patency of the bypass grafts for follow up, particularly in the presence of competitive flow, which may lead to stenosis of bypass grafts.

Small children undergo general anesthesia, while older children can tolerate conscious sedation for the procedure. Patients are heparinized, and an activated clotting time of ~250 seconds is maintained throughout the procedure. An aortic root angiogram prior to selectively engaging coronary arteries may be helpful. This helps guide the operator in choosing the catheter type based on the origin of the coronary arteries and size of the aorta. Additionally, in the setting of severe proximal stenosis, it alerts the operator of potential hemodynamic compromise during coronary angiography or invasive testing of the coronary arteries, particularly in small coronary arteries. If standard curve catheters are not successful in engaging coronary arteries, the catheters can be shaped by hand or with heat to provide an appropriate curve. This may be necessary in particular in early infancy, for whom limited coronary artery curves are available.

Angiography is performed in angled projections. In children and adolescents, biplane angiography in simultaneous orthogonal views is advantageous and can help limit the amount of contrast administered. For the left coronary artery system in particular, the right anterior oblique (RAO)/cranial projection is used to profile the left anterior descending coronary artery. The left anterior oblique (LAO)/caudal projection (also known as the spider view) profiles the left main coronary artery and is helpful for bifurcating lesions or aneurysms that may be seen in this area. It is also helpful to view the circumflex coronary artery. Both RAO and LAO projections can be used to image the right coronary artery system as well. Further complex angulations are beyond the scope of this article, however, may be needed to image specific coronary artery segments. 

## 3. Fractional Flow Reserve

FFR is the ratio of flow in the coronary artery distal to the stenosis compared to the aortic pressure, with a normal value being 1.0. FFR is measured at baseline, followed by provocative testing with adenosine (140 mcg/kg/min) infusion for 3 minutes [3]. Mean FFR is used for assessment for fixed coronary stenosis. A cut off value of ≤0.8 is considered positive for indications for coronary revascularization. There is emerging data in adults that instantaneous wave free-ratio (iFR), which avoids the use of hyperemic agents like adenosine, gives similar diagnostic accuracy compared to FFR with adenosine [4]. This technology has the potential to reduce procedural time, cost, and adverse events associated with medication administration, however, needs further evaluation in children. 

In adults with atherosclerotic coronary artery disease, it has been shown that the addition of FFR is superior to angiography alone to guide percutaneous coronary interventions [5]. The mechanism of coronary stenosis in Kawasaki disease is related to myofibroblastic proliferation, thrombosis, and calcification [6]. Stenosis may occur within the aneurysm, and not infrequently around the margins of the aneurysms. Although this is different from atherosclerosis, FFR has demonstrated to be a reliable marker to assess the severity of coronary stenosis in Kawasaki disease [7]. In children, generally 4–5 Fr catheters are used for FFR measurement. However, in very small children, even 3.3 Fr catheters can be used for this purpose, as the 0.014” wire required can be introduced within a 3.3 Fr catheter without a dampening effect. It is important to remember that if the catheter obstructs the coronary ostium, it should be withdrawn to the aorta for accurate measurements to prevent dampening effects and inaccurate measurements.

## 4. Intravascular Ultrasound

IVUS has increased our understanding of the vascular changes that occur in the coronary artery wall post Kawasaki disease. Long term follow up studies with IVUS have shown persisting abnormal vascular wall morphology and endothelial dysfunction at the site of coronary aneurysms that have regressed in patients with Kawasaki disease [8]. Various degrees of intimal thickening and abnormal vascular response to acetylcholine and isosorbide dinitrate infusion have been noted [8]. The degree of calcification can be quantified using IVUS and can be used in the decision-making towards optimal mode of intervention in the coronary artery. The major drawback of this technology is that it requires the use of 5–6 Fr guiding catheters, and hence it is not routinely performed in small children.

## 5. Optical Coherence Tomography

OCT is an emerging technology with higher spatial resolution than IVUS in visualization of microscopic details of the coronary artery wall, though the depth of imaging is limited [9,10]. Another limitation of this technique includes the need to displace blood within coronary arteries during image acquisition. In children, it can be performed using a 5 Fr guide catheter. A number of findings have been noted with OCT, the most common being intimal hyperplasia, fibrosis and disappearance of the medial layer [11,12]. Although the significance of these findings are not clear, they may correspond with coronary endothelial dysfunction which may put patients at risk of future cardiac events. Further refinements in this technology is required before it can be utilized more extensively in children.

## 6. Treatment

Catheter interventions and bypass surgery both have their merits and drawbacks and complement each other to provide optimal outcomes for children with Kawasaki disease. It is important to emphasize that catheter-based interventions may help palliate significant coronary lesions and help buy time before a bypass graft is required, a strategy that seems logical in select young children. Future options for bypass grafting are generally not jeopardized in these vessels. In some children, catheter interventions may be curative, and in those with multi-vessel disease, a staged approach can be taken while treating the most severe lesions first. However, it is important to recognize that patients undergoing transcatheter interventions as the first procedure may have higher re-intervention rates when compared to those undergoing coronary artery bypass graft (CABG) [13,14]. Generally, coronary lesions that are localized or short segment are amenable to transcatheter interventions, and patients with severe left ventricular dysfunction are best treated with a bypass surgery as the first step [15].

## 7. Indications for Catheter Interventions and Consideration for Coronary Artery Bypass Grafting

Indications for catheter-based interventions should take into consideration institutional or local expertise and involvement of appropriate specialists (i.e., adult interventional cardiologists) when needed. Local surgical expertise should also be taken into important consideration. 

Management of acute thrombosis in the cardiac catheterization laboratory can be performed. If medical therapy fails and there is a need for acute catheter-based thrombolysis, a number of new therapies may be utilized for acute coronary artery thrombolysis depending on institutional experience [16].

Recommendations from experts in Japan have been published for catheter-based treatment of coronary artery stenotic lesions in Kawasaki Disease. Both single vessel and multivessel focal disease can be treated in the cardiac catheterization laboratory. These include any patient with symptoms of ischemia or findings of ischemia on testing. Patients with more than 75% narrowing in the left anterior descending coronary artery, which can be a cause of sudden death, should be considered for revascularization, either catheter-based or surgical. Catheter-based interventions should also be considered for high risk CABG patients. Numerous reports and series of catheter-based therapies have been published, however, data regarding long term outcomes are lacking [15].

Although percutaneous coronary interventions can be performed in children and adolescents with single vessel disease or multivessel focal disease; in those patients with multivessel disease and diminished LV function, CABG may be the preferred first line of treatment [2]. An elegant study from Japan showed excellent long-term results from CABG in children with Kawasaki disease, with the vast majority of patients surviving at long term follow-up with excellent functional status. However, reinterventions were common, stressing the need for close follow-up surveillance [17].

## 8. Balloon Angioplasty

Previous studies have shown that balloon angioplasty is effective for patients with shorter duration of onset from the disease, some indicating that within 6 years of the onset of Kawasaki disease but at >6 years after onset, it is less effective [18,19,20]. Stenotic coronary lesions early in the disease process are usually caused by intimal hypertrophy without significant calcification and are best treated with balloon angioplasty. The diameter of the balloon chosen is equal to or slightly smaller than the normal artery adjacent to the stenosis. A short balloon length is preferred in order to limit its expansion to the affected part of the vessel. The advantages of this mode of therapy is that it can be applied to very small children as well, and all future options for other interventions or surgery are still preserved. 

Post angioplasty, patients with Kawasaki disease need to be watched for development of new coronary aneurysms [18,21]. Although the mechanisms are unclear, they may be related to high pressure balloon angioplasty [18,21]. Hence, it is recommended to keep the maximum balloon pressure below 10 atm [18,21]. Slow inflation and deflation of balloons may be beneficial as well. In cases of complete coronary artery occlusion that are unable to be crossed with a wire, microcatheters can be helpful to cross such lesions prior to angioplasty. In general, due to the above limitations, balloon angioplasty in isolation is usually not an effective treatment modality for these patients and often needs to be performed in addition to rotational angioplasty or stenting.

## 9. Stenting

**Figure 1:**
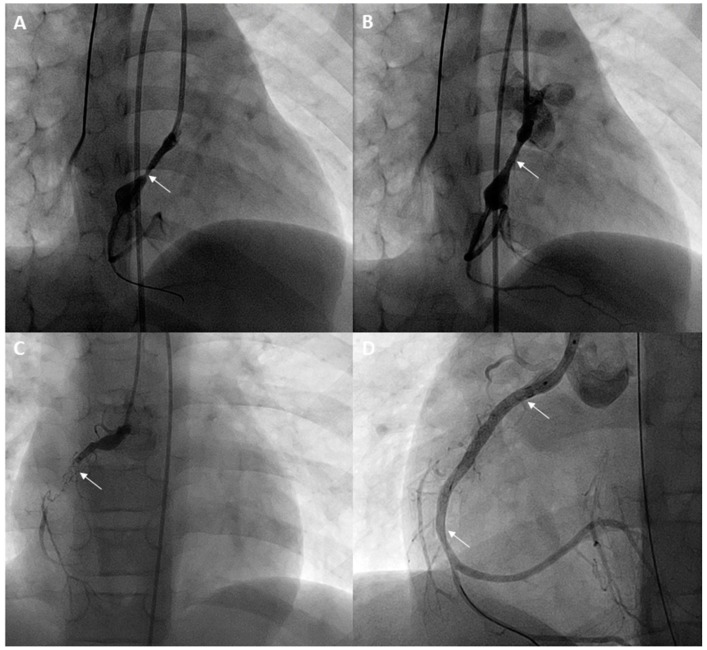
A 7-year-old with a history of Kawasaki disease and giant coronary artery aneurysms of the left main coronary artery and right coronary artery underwent a cardiac catheterization. Fractional flow reserve with adenosine in the right coronary artery (RCA) was 0.74. Stenosis proximal to the aneurysm in the RCA is seen (arrow) (**A**). After placement of a drug-eluting stent, the stenosis has resolved (arrow) (**B**). Two years later, a cardiac MRI showed an inducible perfusion defect in the inferior septum and inferior wall. Angiograms in the RCA showed complete occlusion of the RCA stent (arrow) (**C**). After recanalization and angioplasty, an overlapping stent was placed extending to the distal RCA (lower inferior arrow) (**D**) and an additional proximal stent was also placed (superior arrow) (**D**), reestablishing flow in the RCA.

Children and adolescents with Kawasaki disease who have recurrent stenosis following angioplasty should be considered for stenting (Figure 1). Although bare metal stents have been used for this purpose in the past [18,21,22], drug eluting coronary stents should be used in this era and can generally be introduced using 5 Fr guide catheters as well. These stents have excellent patency rates and have low incidence rates of new aneurysm formation. The diameter of the stent chosen should be equal to the size of the adjacent normal vessel. The shortest length of the stent should be used and care should be taken to avoid jailing any side branches. Following the procedure, stent prophylaxis is necessary. In addition to Aspirin 5 mg/kd/day, clopidogrel 4 mg/kg loading dose is given followed by maintenance with 1mg/kg/day in children for more than 2 months [22], and preferably up to 6 months, based on extrapolation from adult data. Low molecular weight heparin or coumadin should be used if large coronary aneurysms are still present. Although there is lack of long-term data, it may be beneficial to use statins in Kawasaki disease patients with severe coronary artery disease.

## 10. Rotational Ablation

Rotational ablation technology employs a diamond coated burr that rotates at 200,000 rpm to treat coronary artery stenosis and even lesions that are severely calcified [21]. Rotational ablation in addition to stent implantation may yield superior results in calcified lesions. The primary limitation of this technique is that it requires larger arterial access, and hence, it is mainly suited for larger patients [18,21].

## 11. Complications, Limitations, and Future Directions

Although infrequent, complications, such as coronary artery dissection, acute thrombosis, brady arrhythmia, atrio-ventricular block, ventricular tachycardia or fibrillation may occur and can have serious implications, even proving to be fatal. Centers should be equipped to manage such situations and other adverse events. Transcatheter coronary artery interventions can now be performed in small children. However, collaboration with adult cardiologists and surgeons is a very important aspect to move this field forwards. We need to develop equipment focused on this population, such as 5 Fr and smaller catheters with pediatric curves. Further miniaturing the coronary balloon and stent technology will be beneficial for small children. These procedures have a steep learning curve and should be performed in specialized centers with expertise in caring for these children. Multi-institutional registries need to be set up to continually evaluate performance to strive for optimal outcomes.

## 12. Conclusions

Cardiac catheterization has an important role in the evaluation and treatment of children and adolescents with Kawasaki disease. Transcatheter coronary artery interventions can now be performed safely in small children. Optimal outcomes are dependent on a collaborative team approach between cardiologists, cardiac interventionalists, and surgeons. Coronary artery disease in children and adolescents with Kawasaki disease can occur in a myriad of forms and merits an individualized approach to such patients considering the local expertise and availability of resources.
